# Case Report: Paliperidone Palmitate in the Management of Bipolar I Disorder With Non-compliance

**DOI:** 10.3389/fpsyt.2020.529672

**Published:** 2021-01-08

**Authors:** Kanglai Li, Yingtao Liao, Zhihua Yang, Caishuang Yang, Minhua Chen, Xiuhua Wu, Zhaoyu Gan

**Affiliations:** ^1^Very Important Patients Department, The Third Affiliated Hospital of Sun Yat-sen University, Guangzhou, China; ^2^Department of Psychiatry, The Third Affiliated Hospital of Sun Yat-sen University, Guangzhou, China

**Keywords:** bipolar disorder, treatment, long-acting injectable antipsychotics, once-monthly paliperidone palmitate, non-compliance

## Abstract

**Background:** Medication non-adherence is prevalent in patients with bipolar disorder (BD). Long-acting injectable antipsychotics (LAIAs) are widely used to improve compliance with treatment. This study aimed to illustrate the effectiveness, compliance, and safety profile of once-monthly paliperidone palmitate (PP1M), a novel therapeutic LAIA, in the management of bipolar I disorder (BDI).

**Method:** A prospective follow-up was arranged to 11 BDI patients who were prescribed PP1M as monotherapy or adjunctive treatment. Severity of symptoms, disturbing behavior, status of employment, 17-item Hamilton Depression Rating Scale (HAMD-17), and Young Mania Rating Scale (YMRS) were evaluated at the baseline and the endpoint of follow-up. Clinical Global Impression—Bipolar Disorder—Severity of Illness Scale (CGI-BP) and Treatment Emergent Symptom Scale (TESS) were measured at each injection of PP1M. Compliance, relapse or switch, and new hospitalization were monitored through the period of follow-up.

**Results:** The median duration of treatment was 14 months, ranging from 5 to 22 months. The scores (mean ± standard deviation) of HAMD-17, YMRS, and CGI-BP generally decreased from the baseline (16.1 ± 10.3, 30.9 ± 12.6, 5.3 ± 0.7) to the endpoint (7.4 ± 5.7, 3.7 ± 3.2, 2.3 ± 0.7). No disturbing behavior was detected at the endpoint. Neither new hospitalization nor manic/mixed episode occurred during treatment, whereas mild to moderate depressive episodes were reported in three cases. The status of employment of 10 participants (90.9%) was improved, and no new safety concern was detected.

**Conclusion:** PP1M might offer a new valid treatment option in the long-term management of BDI, especially for those with poor compliance with oral medication. However, more studies are needed to further justify such role.

## Introduction

Bipolar disorder (BD) is a common and disabling mental disorder, with a lifetime prevalence of 2.4% in the world (1). The pooled suicide rate in BD is as high as 164 per 100,000 person-years, accounting for 3.4–14% of all suicide death (2). Although the etiology of BD is not yet clear, long-term pharmaceutical treatment is still the most effective methods to ameliorate symptoms and prevent relapses and chronification. However, the high non-adherence rate ranging from 20 to 60% remains as a formidable challenge in the clinical practice for patients with BD (3). For BD patients with current major depressive episode (MDE), our previous report (4) has demonstrated that over 60% of treatment interruption occurred within 12 months after treatment initiation, and that over 40% of treatment discontinuation happened in the first 3 months. Medication non-adherence in BD not only is associated with high risk of recurrence, relapse, and hospitalization but also decreases the rate of remission and recovery (5, 6).

Long-acting injectable (LAI) drug is considered to be one of the most promising strategies to manage medication non-adherence in BD, which has been shown to decrease the risk of suicide attempts and lower the mental health care expenditure (7, 8). Findings from real-world study suggest that LAI antipsychotics ensure a better medication adherence for patients with schizophrenia or BD than oral antipsychotics (9). However, few LAI second-generation antipsychotics have been approved for the treatment of BD currently. To date, only LAI risperidone and LAI aripiprazole have received approval by the Federal Drug Administration for this indication (3). As a long-active formulation of the active metabolite of risperidone, paliperidone palmitate (PP1M) has demonstrated its efficacy and safety in the long-term management of schizophrenia. Paliperidone extended-release (ER) has been proven to be effective and safe to treat acute manic or mixed episode, thus recommended by the 2018 Canadian Network for Mood and Anxiety Treatments (CANMAT) to be a first-line monotherapy option for initial mania treatment (10–12) and a second-line therapy option for maintenance treatment (13, 14). Once-monthly PP1M, as monotherapy or adjunctive therapy, is also shown to significantly delay psychotic, depressive, and/or manic relapses in patients with schizoaffective disorders (15). That is to say, the efficacy of the chemical component of paliperidone in the management of bipolar I disorder (BDI) has been justified in previous studies.

Based on these facts, we hypothesize that PP1M is therapeutically promising in the long-term management of BDI. However, there are few evidences about PP1M in BDI patients. Thus, in this study, we followed up patients with BDI who were treated with PP1M, aiming to evaluating the effectiveness, compliance, and treatment-emergent adverse events (TEAEs) of PP1M among these cases.

## Methods

### Study Design and Subjects

This was an open-label, one-arm, prospective observational study. All the participants in this study came from patients who sought medical help for BD at the psychiatric department of the Third Affiliated Hospital of Sun Yat-sen University between July 2017 and December 2018. The diagnosis of BD and mental comorbidities was made according to the Diagnostic and Statistical Manual of Mental Disorders, Fourth Edition, Text Revision (DSM-IV-TR) based on the subjects' history of present illness and routine mental examination. The inclusion criteria of this study were: (1) aged 16–65 years, (2) met the DSM-IV-TR criteria for BDI, (3) responded well and had good tolerance to oral risperidone or paliperidone ER treatment, and 4) had a history of non-compliance with oral psychopharmaceutical treatment or claimed unwilling to take oral medication every day or having difficulty in complying with oral psychopharmaceutical treatment after being discharged from hospital. Participants who had one of the following conditions were excluded: (1) unable or refused to provide written informed consent, (2) currently comorbid with severe physical illness or intellectual disability that hindered the participants from completing the required mental evaluation, and (3) unable to be followed up in the long haul.

Once the patients met the inclusion criteria, a face-to-face interview was arranged by the study team member in charge of participant recruitment. Information about the off-label use of PP1M in BD, including the potential benefit and risk of this treatment, was told to all the potential participants and their custodians. Once agreement was achieved, written informed consent signed by both the participants and one of their custodians was provided. This study was reviewed and approved by the Clinical Research Ethics Committee of the Third Affiliated Hospital of Sun Yat-sen University.

### Treatment of the Participants

PP1M was prescribed as monotherapy or adjunctive treatment by the participants' treating psychiatrists according to the patients' condition and willingness. Other psychopharmaceutical treatment was permitted before the administration of PP1M. Except the first two doses of PP1M were fixed (the first dose was 150 mg, and the second was 100 mg), the other doses of PP1M and the duration of PP1M treatment were flexibly decided by the participants' treating psychiatrists according to the patients' condition. In addition, any combined treatment, if necessary, was also decided by the treating psychiatrists.

### Evaluation of Baseline Clinical Characteristics of BD

General sociodemographic and clinical characteristics, including the duration of illness, number of previous affective episodes, and duration of current episode, were collected *via* a questionnaire designed by the investigators. Physical comorbidities were collected by reviewing the patients' previous medical history and medical records stored in the electronic medical system. Severity of the illness were evaluated by the 17-item Hamilton Depression Scale (HAMD-17) (16), the Young Mania Rating Scale (YMRS) (17), and the Clinical Global Impression—Bipolar Scale (CGI-BP) (18). Psychotic features were assessed by evaluating whether the participants demonstrated any of the psychotic symptoms, including hallucination, delusion, or disorganized behavior, in the course of illness.

### Measurement of Efficacy and Safety Profile of the Treatment

The efficacy of treatment was measured from the following aspects: compliance with pharmaceutical treatment, symptomatic improvement, prevention of relapse or switch to another polarized affective episode, prevention of hospitalization, control of disturbing behavior, and restore of employment.

Symptomatic improvement was evaluated by comparing the difference in the scores of HAMD-17, YMRS, and CGI-BP between the baseline and the endpoint. CGI-BP was also measured at each injection of PP1M. Relapse or switch to the other polarized affective episode and hospitalization were monitored through the period of follow-up. Disturbing behavior was defined as any behavior that might threaten the patient's life, properties, and public order, including suicide attempt, fighting, destroying properties, harmful use of psychoactive substance, and so on. Status of employment was divided into three categories according to the participant's attendance in work or school: unemployment (which meant no attendance in work or school), part-time (which meant being partly involved in work or school), and full-time (which meant being fully involved in work or school). Disturbing behavior and status of employment were assessed at the baseline and the endpoint based on the past 1-month-related information. The safety of treatment was measured by Treatment Emergent Symptom Scale (TESS) at each study visit as a routine clinical assessment to assess any TEAEs. At the same time, any available results of laboratory examinations conducted during the period of follow-up would be reviewed to detect any potential treatment-related safety problem. The tolerability of PP1M was assessed by asking the reasons of treatment discontinuation if it happened.

### Definition of the Final Treatment Outcome

The final treatment outcome is divided into three categories (19): relapse or switch, response, and remission. Relapse or switch is defined as a return or switch to the full syndrome criteria of an episode of mania, mixed episode, or depression following a remission of any duration. If relapse or switch is detected at any time during the period of follow-up, the final treatment outcome is categorized as relapse or switch, regardless of the scores of HAMD-17 or YMRS at the endpoint. Remission is defined as HAMD-17 <8 and YRMS <8 at the endpoint, whereas no relapse or switch is detected during the period of follow-up. Response is defined as the 50% reduction in the scores of HAMD or YRMS from the baseline to the endpoint while the other pole cannot be significantly worsened.

### Follow-Up

The follow-up started from the first injection of PP1M. The follow-up visit was arranged at each injection of PP1M. If the PP1M treatment was not discontinued, the follow-up would go on until June 30, 2019. The final injection before treatment discontinuation or the last injection before June 30, 2019 was considered as the endpoint of the follow-up.

### Statistical Analysis

The prevalence of discrete variables was calculated. Mean and standard deviation of normally distributed data were analyzed, whereas median and range from minimum to maximum were reported for non-normally distributed variables. Difference in the final treatment outcome between the groups was compared using Chi-square test. Mann–Whitney *U*-test was performed to compare the duration of follow-up between the different groups. The distribution of the scores of CGI-BP for all the participants was plotted using Microsoft Office Excel 2007 (Microsoft Corporation, Redmond, WA, United States). The results were considered significant at *P* < 0.05. All data were analyzed using commercial statistical package SPSS 19.0 (SPSS, Inc., Chicago, IL, United States).

## Results

### Demographic and Clinical Characteristics of the Participants

Eleven patients with BDI were eligible for this study; 10 (90.9%) of them were recruited when being admitted to hospital. The demographic and clinical characteristics of the participants are listed in Table 1. Of the 11 participants, 5 (45.4%) were female, their age ranged from 16 to 63 years with a median of 33 years, and 6 (54.5%) were married. At the baseline, all the participants were in manic or mixed episodes, 7 (63.6%) presented with psychotic features, 4 (36.4%) experienced recent suicide attempts, 4 (36.4%) were comorbid with abnormal glucose or lipid metabolism, 3 (27.3%) had psychoactive substance abuse, 2 (18.2%) were with thyroid diseases, their duration of illness ranged from 1 to 10 years with a median of 4 years, and 7 (63.6%) had experienced at least one depressive episode, whereas all the participants had experienced at least one (hypo)manic/mixed episode.

**Table 1:** The demographic and clinical characteristics of all the participants.

**No**.	**Age**	**Sex**	**Marriage status**	**Current episode**	**Psychotic feature**	**Comorbidity**	**DI (y)**	**NDE**	**NME**	**DCE (m)**
1	37	F	Divorced	Manic	No	None	1	1	3	3
2	33	M	Married	Mixed	Yes	Suicide attempt, nicotine dependence	9	4	2	108
3	16	M	Unmarried	Mixed	Yes	Suicide attempt	3	1	1	2
4	27	M	Married	Manic	Yes	Obese, hepatic adipose infiltration, Hashimoto's thyroiditis	9	0	3	12
5	63	F	Married	Manic	Yes	Hepatic adipose infiltration, hyperlipidemia, left middle cerebral artery occlusion	2	0	3	0.25
6	20	F	Unmarried	Mixed	Yes	Suicide attempt, alcohol abuse, impaired glucose tolerance	8	2	1	6
7	26	F	Unmarried	Mixed	Yes	Acquired renal cyst	9	5	4	0.07
8	35	M	Married	Manic	Yes	Suicide attempt	10	2	1	0.5
9	38	M	Married	Mixed	No	None	3	3	3	4
10	33	F	Married	Manic	No	Arrhythmia, pneumonia, heart failure	4	0	3	0.17
11	23	M	Unmarried	Mixed	No	Diabetes, hypothyroidism, nicotine dependence	3	0	1	48

*DI, duration of illness; NDE, number of depressive episode; NME, number of (hypo)manic or mixed episode; DCE, duration of current episode*.

### Treatment Information of the Participants

The detailed information on the treatment of all the participants is listed in Table 2. Nine (81.8%) had received pharmaceutical treatment before the recruitment, with the time from the first dose of medication to recruitment ranging from 1 to 120 months. At the acute phase of treatment, only 3 (27.3%) participants were treated with PP1M monotherapy, and the rest received combined treatment, including 2 (18.2%) with modified electroconvulsive therapy (MECT). At the endpoint, 6 (54.5%) participants maintained treatment with PP1M monotherapy, and the rest received concomitant lithium, alprazolam, or fluoxetine with PP1M treatment.

**Table 2:** Treatment of all the participants.

**No**.	**Clinical setting**	**Medications before PP1M**	**TR (m)**	**Dose of PP1M3 (mg eq)**	**Dose of PP1Mf (mg eq)**	**Concomitant treatment at the acute phase**	**Duration of hospitalization (d)**	**Concomitant medications at the endpoint**	**Discontinuation of PP1M**	**Reason for discontinuation**	**TD (m)**
1	Inpatient	Quetiapine, sodium valproate, estazolam	1	100	75	Lithium ER 0.6/d, benzhexol 2 mg bid, alprazolam 0.4 mg bid, MECT 6 courses	17	Lithium 0.9/d, alprazolam 0.4 mg/d	Yes	Depressive episode	8
2	Inpatient	Olanzapine 10 mg/d, divalproex sodium 1.0/d	3	100	75	Lithium ER 0.9/d, benzhexol 2 mg bid, alprazolam 0.4 mg qn	7	Lithium 1.2/d, alprazolam 0.4 mg prn	Yes	Economic problem	10
3	Inpatient	None	0	150	75	Lithium 0.9/d, Seroquel 0.05 qn	16	Lithium 0.9/d, fluoxetine 10 mg/d	Yes	Decreased motivation	5
4	Inpatient	Aripiprazole 10 mg/d	68	150	100	None	18	None	No		
5	Inpatient	Risperidone, PP1M, paliperidone ER, sodium valproate, benzhexol	60	100	75	Lithium ER 0.9/d, quetiapine 100 mg/d, divalproex sodium 1.0/d, ziprasidone injection 40 mg/d, clonazepam 1 mg/d, alprazolam 0.4 mg/d, hydroclopidogrel 75 mg/d, atorvastatin calcium 10 mg/d	19	Hydroclopidogrel 75 mg/d, atorvastatin calcium 10 mg/d	Yes	Self-decided because of decreased energy	14
6	Inpatient	None		100	150	Quetiapine 400 mg/d, oxcarbazepine 0.6/d, lithium ER 0.9/d, MECT 9 courses	16	None	No		
7	Inpatient	Lamotrigine 100 mg/d, clozapine 300 mg/d, divalproex sodium 0.75/d, lithium 1.0/d, aripiprazole, risperidone 4 ml/d	34	100	100	Clozapine 50 mg/d, aripiprazole 5 mg/d, lithium 0.5/d	9	None	Yes	Self-decided for plan to get pregnant	15
8	Inpatient	Clozapine	120	100	75	Lithium ER 1.2/d, clonazepam 2 mg/d	14	Lithium ER 0.9/d	Yes	Depressive mood	6
9	Inpatient	Paliperidone ER, sodium valproate, escitalopram	36	100	75	None	16	Lithium 0.6/d	No		
10	Inpatient t	Divalproex sodium 0.75/d, quetiapine 500 mg/d	36	100	75	Divalproex sodium 0.75/d, quetiapine 600 mg/d, haldol 20 mg/d (im), clozapine 150 mg/d	14	Metformin 1.0/d, atorvastatin calcium 10 mg/d	No		
11	Outpatient	Paliperidone ER	5	150	150	None	NA	None	No		

*TR, time from the first dose of medication to recruitment; PP1M3, the third injection of one-monthly paliperidone palmitate (PP1M); PP1Mf, the final injection of one-monthly paliperidone palmitate (PP1M); TD, time to discontinuation; NA, not applicable*.

### Compliance With PP1M Treatment

As seen in Table 2, up until June 30, 2019, 6 (54.5%) participants discontinued the PP1M treatment. None of them was caused by adherence issue. Among the discontinuations, 4 (36.4%) were attributed to efficacy reason, and 2 (18.2%) were for personal reasons that were not associated with the medication itself. The median duration of treatment was 14 months, ranging from 5 to 22 months.

### Treatment Outcome of PP1M

**Figure 1:**
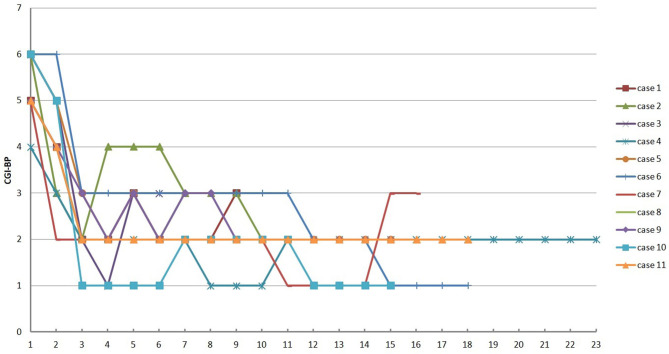
The distribution of CGI-BP of all the participants over each injection of PP1M.

The treatment outcome of the 11 participants is illustrated in Table 3. The scores (mean ± standard deviation) of HAMD-17, YMRS, and CGI-BP generally decreased from the baseline (16.1 ± 10.3, 30.9 ± 12.6, 5.3 ± 0.7) to the endpoint (7.4 ± 5.7, 3.7 ± 3.2, 2.3 ± 0.7). Figure 1 further illustrates the change of the scores of CGI-BP over each injection of PP1M. No new hospitalization happened to any of the participants during the period of PP1M treatment. Mild to moderate depressive episode (8 < HAMD-17 <24 or 2<CGI-BP <5) was detected among 3 cases (27.3%) during the PP1M treatment, whereas no (hypo)manic episode was seen during the same period. At the endpoint, no disturbing behavior was detected, whereas it was seen in 10 (90.9%) cases at the baseline. After PP1M treatment, only 1 case's status of employment (9.1%) deteriorated from part-time schooling to suspension of schooling, the other cases' status of employment got improvement, including 4 (45.4%) from unemployment to full-time work, 4 (45.4%) from unemployment to part-time work, and 2 (18.2%) from part-time work to full-time work.

**Table 3:** Treatment outcome of all the participants.

**No**.	**Baseline**						**DF (m)**	**Relapse or switch^**b**^**	**Hospitalization^**b**^**	**Endpoint**				
	**HAMD-17**	**YMRS**	**NPH**	**CGI-BP**	**SE^**a**^**	**DB**				**DB^**b**^**	**HAMD-17**	**YMRS**	**CGI-BP**	**SE^**a**^**
1	10	35	2	5	0	Running away from home for no reason	8	1	0	0	16	2	3	1
2	31	31	0	6	0	Aggressive behavior	10	0	0	0	7	4	2	2
3	28	14	0	5	1	Suicide attempt	5	1	0	0	15	7	3	0
4	9	25	1	4	0	Lavish spending	22	0	0	0	3	5	2	2
5	10	44	1	6	0	Agitated behavior	14	1	0	0	6	2	2	1
6	29	31	0	6	1	Binge drinking, suicide attempt, promiscuous sex	18	0	0	0	1	1		2
7	19	25	2	5	0	Fighting against her mother	15	0	0	0	8	11	3	2
8	5	51	0	6	0	Binge drinking, suicide attempt, masturbation in public	6	1	0	0	15	3	3	1
9	17	11	0	5	1	No	14	0	0	0	6	0	2	2
10	0	46	2	6	0	Aggressive behavior	14	0	0	0	0	1	1	2
11	19	27	0	5	0	Lavish spending, aggressive behavior	17	0	0	0	4	5	2	1

*HAMD-17, the 17-item Hamilton Depression Scale; YMRS, the Young Mania Rating Scale; NPH, number of previous hospitalization; SE, status of employment; DB, disturbing behavior; DF, duration of follow-up*.

a *0, unemployment; 1, part-time employment; 2, full-time employment*.

b *0, no, 1, yes*.

### Comparison of the Final Treatment Outcome Between the PP1M Monotherapy Group and the PP1M Combined Treatment Group

According to whether other psychopharmaceutical or MECT treatment was combined with PP1M at the baseline and at the endpoint, treatment group was divided into the initial/final PP1M monotherapy group and the initial/final PP1M combined therapy group. Chi-square test was performed to compare the final treatment outcome between the above-mentioned two groups, respectively. Duration of follow-up was also compared between the two groups using Mann–Whitney U test. No significant difference in the final treatment outcome was found between the initial/final PP1M monotherapy group and the initial/final PP1M combined treatment group (*P* > 0.05). Duration of follow-up (median, minimum–maximum) was significantly longer in the final PP1M monotherapy group than in the final PP1M combined group (16.14–22 vs. 8.5–14) (*P* = 0.009), but it did not significantly differ between the initial PP1M monotherapy group than in the initial PP1M combined group (17.14–22 vs. 12.5–18) (*P* > 0.05).

### Safety of PP1M Treatment

As seen in Table 4, the most common TEAE during PP1M treatment was sedation, reaching 63.6%. Weight gain came next, with a prevalence of 54.5%. The third rank TEAEs were prolactin-related adverse events, decreased energy, and insomnia, which shared the same prevalence of 45.5%. The other less common TEAEs included depressive mood (27.3%), extrapyramidal symptoms (EPS) (18.2%), sickness (9.1%), and injection-site pain (9.1%).

**Table 4:** The prevalence of adverse events during the treatment.

**No**.	**Adverse events (AE)**^****a****^								
	**EPS**	**Prolactin-related AE**	**Decreased energy**	**Sedation**	**Sickness**	**Weight gain**	**Depressive mood**	**Insomnia**	**Injection-site pain**
1	1	1	1	1	1	0	1	1	0
2	1	0	0	0	0	0	0	1	0
3	0	1	1	1	0	1	0	1	0
4	0	1	0	0	0	1	0	0	0
5	0	0	1	1	0	0	0	0	0
6	0	0	0	1	0	0	0	0	0
7	0	1	0	0	0	1	0	1	0
8	0	0	1	1	0	1	1	1	0
9	0	0	1	1	0	1	1	0	0
10	0	1	0	1	0	1	0	0	1
11	0	0	0	0	0	0	0	0	0
The prevalence of AE (%)	18.2	45.5	45.5	63.6	9.1	54.5	27.3	45.5	9.1

*EPS, extrapyramidal symptoms*.

a *1, occurred; 0, not occurred*.

## Discussion

In this prospective study, we enrolled 11 BD patients who were prescribed PP1M as monotherapy or adjunctive treatment. The scores of HAMD-17, YMRS, and CGI-BP generally decreased from the baseline to the endpoint. All the disturbing behavior occurring at the entry was under control after treatment. Neither new hospitalization nor manic/mixed episode occurred during the period of PP1M treatment, whereas mild to moderate depressive episodes were reported in three cases. The status of employment improved among most of the cases, and no new safety concern was detected.

Speaking in terms of pharmacokinetics (20), PP1M provides consistent therapeutic plasma concentration over 4 weeks, eliminating the need for daily oral medication, and therefore it helps to improve the treatment adherence. On contrary to the fact that most of the participants showed poor compliance with pharmaceutical treatment before PP1M was prescribed, all the participants in this study complied with the PP1M treatment as scheduled during the period of follow-up. This study supports this view in BD patients with poor compliance with pharmaceutical treatment, which is consistent with a previous case report that PP1M significantly improved the adherence to treatment among three psychotic BD patients who were not compliant with oral medication (21). Although previous randomized controlled trials (RCTs) did not find that long-acting injectable antipsychotics (LAIAs) were superior to oral corresponding medication in terms of compliance and re-hospitalization rates, the highly compliant study populations in these studies might contribute to this negative result. In this study, all the participants had a history of non-compliance with medication or high risk of future non-compliance. Furthermore, some of them were undergoing a very severe manic episode, some experienced a recent suicide attempt, and others had comorbid psychoactive substance abuse. All these clinical characteristics were proven to be associated with non-compliance (22–26), preventing them from recruitment to any RCT study for ethical reasons. Therefore, our study adds to the evidence that PP1M may show advantage in treating BD patients with non-compliance, which usually could not be provided by RCT studies. In addition, PP1M seems to have some efficacy in the management of psychotic or non-psychotic BD in our study, which is consistent with previously demonstrated efficacy of paliperidone ER and PP1M in patients with current manic or mixed episodes, schizoaffective disorders (15), or psychotic BD (21). However, depressive episode was detected in three cases, suggesting that PP1M may be less effective in the prevention of depression, which is in line with the idea that depot neuroleptics showed better efficacy in preventing mania than depression (27). Combined with the fact that manic symptoms are more associated with non-compliance than depressive symptoms (28), BD with a predominance of manic symptoms may be more suitable to use PP1M.

The pattern of TEAEs in this study was similar to those previously reported in studies of PP1M in schizophrenia (29, 30), schizoaffective disorder (15, 31, 32), and BD (21). In addition, no new safety concern was found, and no case was withdrawn from the PP1M treatment because of safety concern, suggesting that PP1M is safe and tolerable in the long-term management of BD.

However, careful interpretation of the results from this study is needed because of some limitations. First, only 11 participants were included in this study. Such small sample size might dramatically weaken the capacity of this study's findings for generalization. Since most of the participants had a prominence of mania and were highly compliant with oral medication treatment, we cannot tell whether BD with a prominence of depression and high compliance would benefit from the PP1M treatment. Second, this study was an open-label, single-armed study, and combination of other treatment was allowable on several cases, especially at the acute phase. Third, in terms of clinical assessments, disturbing behavior, social function, and compliance were not usually assessed with standardized rating scale. Fourth, laboratory examinations were not always performed regularly, making some safety problem undetected. Large sample size, prospective standard controlled studies are needed in the future. Although with these limitations, the results presented here still show evidence that PP1M may be effective, safe, and tolerable in the long-term management of BD as monotherapy or adjunctive treatment. This effectiveness was demonstrated by the great improvement in the compliance to treatment, general decrease in the scores of HAMD, YMRS, and CGI, reduction in the number of mood episodes and hospitalization, and improvements in behaviors and social functions. More importantly, this effectiveness was unlikely to be due to placebo effect since 9 of the 11 participants had failed to response to active psychopharmaceutical treatment before. Nor could this effectiveness be completely attributed to the combined treatment, since no significant difference was found between the initial/final PP1M monotherapy group and the initial/final PP1M combined treatment group, and some combined treatment had been tried on some cases but failed to be complied with.

In summary, this small sample size study shows evidence that PP1M may be beneficial in the long-term management of BDI with a prominence of manic symptoms as monotherapy or adjunctive therapy. Especially in those with poor compliance with oral medication, PP1M may provide a new valid treatment option.

## Data Availability Statement

All datasets generated for this study are included in the article/supplementary material.

## Ethics Statement

The studies involving human participants were reviewed and approved by the Clinical Research Ethics Committee of the Third Affiliated Hospital of Sun Yat-Sen University. Written informed consent to participate in this study was provided by the participants' legal guardian/next of kin. Written informed consent was obtained from the individual(s) for the publication of any potentially identifiable images or data included in this article.

## Author Contributions

ZG: conception and design. ZG and XW: recruitment of participants and clinical measurement. CY, ZY, MC, and YL: management of treatment and follow-up. CY, ZY, and MC: collection and management of data. XW, CY, ZY, MC, and KL: analysis and interpretation of data. KL and YL: drafting the article. ZG: revising it for intellectual content. All authors contributed to the article and approved the submitted version.

## Conflict of Interest

The authors declare that the research was conducted in the absence of any commercial or financial relationships that could be construed as a potential conflict of interest.
